# Social feedback processing from early to late adolescence: influence of sex, age, and attachment style

**DOI:** 10.1002/brb3.251

**Published:** 2014-07-23

**Authors:** Pascal Vrtička, David Sander, Brittany Anderson, Deborah Badoud, Stephan Eliez, Martin Debbané

**Affiliations:** 1 Department of Social Neuroscience, Max Planck Institute for Human Cognitive and Brain SciencesLeipzig, Germany; 2 Center for Interdisciplinary Brain Sciences Research, Psychiatry and Behavioral Sciences, Stanford UniversityStanford, California; 3 Swiss Center for Affective Sciences, University of GenevaGeneva, Switzerland; 4 Laboratory for the study of Emotion Elicitation and Expression, Department of Psychology, University of GenevaGeneva, Switzerland; 5 Adolescence Clinical Psychology Research Unit, Faculty of Psychology and Educational Sciences, University of GenevaGeneva, Switzerland; 6 Office Médico-Pédagogique Research Unit, Department of Psychiatry, University of Geneva School of MedicineGeneva, Switzerland; 7 Department of Genetic Medicine and Development, University of Geneva School of MedicineGeneva, Switzerland; 8 Research Department of Clinical, Educational and Health Psychology, University College LondonLondon, UK

**Keywords:** Adolescence, age, attachment style, fMRI, sex, social feedback processing

## Abstract

**Objective:**

The establishment of an accurate understanding of one's social context is a central developmental task during adolescence. A critical component of such development is to learn how to integrate the objective evaluation of one's behavior with the social response to the latter—here referred to as social feedback processing.

**Case report:**

We measured brain activity by means of fMRI in 33 healthy adolescents (12–19 years old, 14 females). Participants played a difficult perceptual game with integrated verbal and visual feedback. Verbal feedback provided the participants with objective performance evaluation (won vs. lost). Visual feedback consisted of either smiling or angry faces, representing positive or negative social evaluations. Together, the combination of verbal and visual feedback gave rise to congruent versus incongruent social feedback combinations. In addition to assessing sex differences, we further tested for the effects of age and attachment style on social feedback processing. Results revealed that brain activity during social feedback processing was significantly modulated by sex, age, and attachment style in prefrontal cortical areas, ventral anterior cingulate cortex, anterior insula, caudate, and amygdala/hippocampus. We found indication for heightened activity during incongruent social feedback processing in females, older participants, and individuals with an anxious attachment style. Conversely, we observed stronger activity during processing of congruent social feedback in males and participants with an avoidant attachment style.

**Conclusion:**

Our findings not only extend knowledge on the typical development of socio-emotional brain function during adolescence, but also provide first clues on how attachment insecurities, and particularly attachment avoidance, could interfere with the latter mechanisms.

## Introduction

A teenage student scoring the highest grade of her class at a math exam might expectedly receive public praise from the teacher while being exposed to hostile looks from some classmates. In the rising complexity of the adolescent's social world (Erikson 1968; Steinberg and Silverberg 1986), the same good objective performance can entail positive, and thus congruent, or negative, and therefore incongruent, social feedback. One of the adolescent's developmental tasks consists in learning how to integrate objective feedback of a performed behavior—that is, success versus failure—with the social evaluation of such behavior—that is, emotional facial expressions representing social support versus disapproval—which we refer to as *social feedback processing*. From a neuroscience perspective, we may ask how the adolescent brain differentially activates to social feedback that is congruent versus incongruent with regard to one's objective performance evaluation.

Over the last decade, a number of studies in the field of social cognitive affective neuroscience have been conducted to elucidate the neural substrates of socio-emotional processing during adolescence (for recent reviews, see e.g., Blakemore 2008; Crone & Ridderinkhof, 2011; Decety et al. 2011; Pfeifer and Blakemore 2012). In so doing, various experimental paradigms assessing face identity and facial emotion perception, mental state representation/theory of mind (ToM), performance in economic games, moral judgment, empathy, or anticipation of/reaction to social evaluation by peers have been employed (Moriguchi et al. 2007; Burnett et al. 2009; Guyer et al. 2009; Forbes et al. 2010, 2011; Gunther Moor et al. 2010; Sebastian et al. 2011, 2012; Pfeifer et al. 2013). These investigations revealed important insights into socio-emotional brain development during adolescence. Yet, to the best of our knowledge, they did not specifically test for social feedback processing as defined above—that is, the integration of objective performance feedback with social evaluation of the latter. It therefore appears that these paradigms were not designed to examine the participants' ecological response *while processing* social evaluations of their objective performance. Investigating such a timeframe might be critical to identify potential biases in social information processing, and furthermore inform how adolescents might *react to* and *learn from* different kinds of social input.

**Figure 1:**
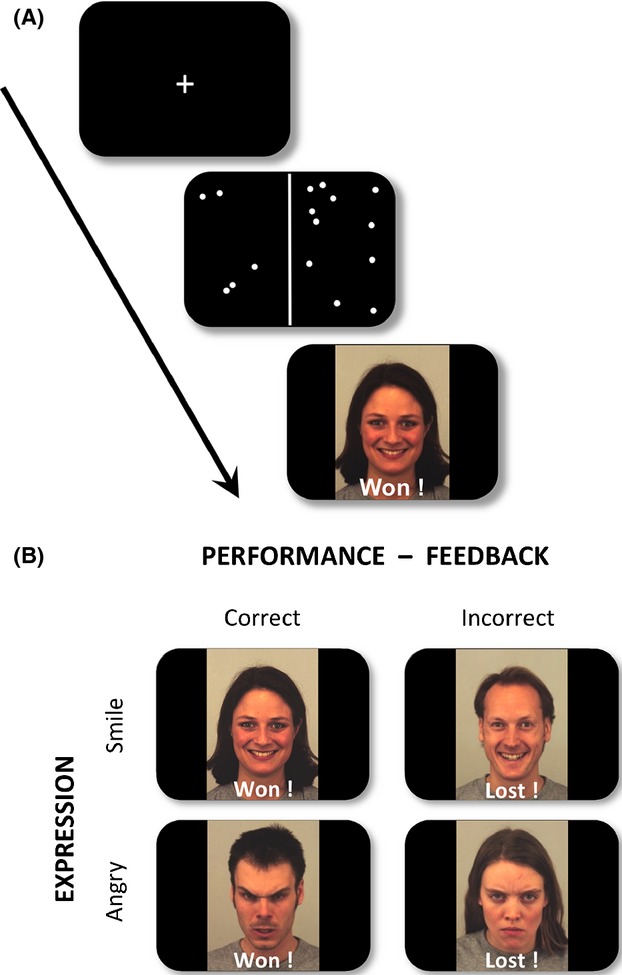
(A) Illustration of the paradigm. Participants first saw a central fixation cross, followed by the dot counting task (0.5 s), where they had to indicate which side of the screen contained more dots (right vs. left). Following each response, a visual feedback was shown (1.5 s), composed of a word together with a face. (B) Illustration of the four different feedback conditions. Two socially “congruent” (Smiling Face on WON trials, Angry Face on LOST trials) and two socially “incongruent” (Smiling Face on LOST trials, Angry Face on WON trials) combinations were possible. Four different face identities (two female and two male) were used in each of these four conditions. See Experimental task section in Methods for further details.

In this study, we used a functional magnetic resonance imaging (fMRI) paradigm specifically targeting brain responses underlying the integration of objective performance feedback with its social evaluation (see Methods and Vrticka et al. 2008), and for the first time applied it to typically developing adolescents. Our research design involved a difficult perceptual game, during which each trial was followed by an objective performance feedback, combined with its social evaluation. In roughly half of cases, objective performance and social evaluation combinations were congruent; in the remaining trials, combinations were incongruent (see Fig. 1 and Methods). By directly contrasting congruent versus incongruent trials, and by further decomposing the found patterns into their respective subcomponents, we could obtain a detailed measure of brain responses to social feedback in adolescents.

In addition to assessing healthy brain function during social feedback processing in adolescence, this study also aimed at examining the influence of potential moderator variables, including sex, age, and attachment style.

Although sex differences in socio-emotional processing have repeatedly been postulated and are generally accepted in folk psychology, empirical fMRI data available up to date remain limited and predominantly report on adult samples (see e.g., Domes et al. 2010; Blakemore 2012). In turn, available behavioral and genetic evidence suggests that female adolescents may be significantly more prosocial than males (Gregory et al. 2009). This study sought to investigate potential sex differences in adolescents' brains potentially related to prosocial behavior by means of differential activation to congruent versus incongruent social feedback.

We further aimed at examining how age could relate to brain responses observed during the processing of social feedback. It is generally understood that adolescence is marked by increasing self-consciousness, perspective taking, and self-regulation skills, as well as stronger prosocial tendencies (van den Bos 2013). The exact effects of age on the neural activation sustaining specific dimensions of social cognition are starting to emerge as a central point of inquiry in developmental cognitive neuroscience. Recent evidence suggests that the (ventro) medial prefrontal cortex and temporal cortical areas (including the temporo-parietal junction) represent regions undergoing age-related changes associated with the above-mentioned socio-emotional functions during adolescence (Blakemore 2012; Pfeifer et al. 2013; Somerville 2013). Along the same lines, other recent research suggests that adolescence is marked by age-related increases in the sensitivity to others' perspectives, specifically regarding trust/cooperation and distrust/unfairness (Fett 2014). Such increased sensitivity may not only involve increased attention devoted to the dimensions of trustworthiness, but also moderate prosocial tendencies in case of conflict.

Finally, we were particularly interested in investigating the influence of individual differences in attachment style with regard to congruency of social feedback processing during adolescence. Attachment measures typically report on basic dimensions (security, avoidance, anxiety) of an individual's characteristic interpersonal schemas (Vrticka and Vuilleumier 2012). These dimensions are thought to be shaped by early relationships (Bowlby 1968; Mikulincer and Shaver 2007), and critically, they are susceptible to exert a top-down effect on socio-emotional information processing (Vrticka and Vuilleumier 2012). Generally speaking, whereas attachment avoidance is usually associated with deactivating strategies to keep the attachment system in a low activation state, attachment anxiety is linked to hyperactivating strategies characterized by sustained strong activation of the attachment system. Preliminary evidence from behavioral and neuroimaging data acquired in adults supports these notions (Vrticka et al. 2008, 2012a,b; Donges et al. 2012; Poore et al. 2012; Vrticka and Vuilleumier 2012). Beyond these global patterns of altered responding, two specific characteristics of attachment avoidance and anxiety are of particular interest. On one hand, attachment avoidance seems to entail a decrease of reward-related neural activity as well as behavioral responding to usually strongly positively valenced (mutual) social situations. On the other hand, attachment anxiety seems to especially increase brain and behavioral responses to scenarios involving social punishment and/or exclusion (see e.g., Vrticka et al. 2008, 2012b; Strathearn et al. 2009; DeWall et al. 2012; Donges et al. 2012; Poore et al. 2012). To the best of our knowledge, the influence of attachment insecurities on adolescent socio-emotional brain function has yet to be explored; therefore, a specific focus on interactions between attachment dimensions and congruency of social feedback processing may be a relevant starting point of neuroscientific inquiry.

On the basis of the above-mentioned literature, we set to test brain activation contrasts relating to (1) incongruent versus congruent social feedback processing during adolescence in general; (2) sex effects on social feedback processing; (3) age effects on social feedback processing; and (4) the effects of attachment insecurity dimensions on social feedback processing. In line with previous work performed in adults, we expected these contrasts to reveal differential activation in an extended social cognition and affective processing network, including—among others—the anterior cingulate cortex, superior temporal sulcus, as well as reward- and self-relevance processing areas.

## Methods

### Participants

Participants were native French-speaking adolescents attending schools in the city of Geneva, Switzerland. Inclusion criteria were age (12–19 years) and right-handedness, and exclusion criteria were clinical levels of internalizing/externalizing symptoms (assessed using Youth and Adult Self-Reports [YSR (Achenbach 1991); ASR (Achenbach and Rescorla 2003)]—clinical levels were defined as a score on either dimension above 65), and known neurogenetic or psychiatric disease. Parental consent for adolescents under age 18 was obligatory to participate in the study. The analysis included 33 healthy participants (14 females). Written informed consent was obtained from participants and their parents under protocols approved by the Institutional Review Board of the Department of Psychiatry of the University of Geneva Medical School. Complete demographics (including personality questionnaire scores) are summarized in Table 1.

**Table 1:** Distribution of continuous measures (sorted by age, ascending). EXT, externalizing, INT, internalizing, AX, attachment anxiety, AV, attachment avoidance, MIN, minimal value, MAX, maximal value, AVG, average, STDEV, standard deviation, Z, Z-score, ZC, corrected Z-score (see below). One participant had missing attachment avoidance and anxiety scores. Another participant had a too low EXT score (MIN-Z = −3.15), and another participant had a too high AV score (MAX-Z = 3.85). These three participants were therefore removed for correlational data analysis (*N* = 30). Maximum and minimum Z-scores (mean = 0 and standard deviation = 1) for *N* = 30 participants are summarized in the table under MAX-Z(C) and MIN-Z(C)

Sex	Age	EXT	INT	AX	AV
Male	12.38	52	42	8	19
Male	12.47	46	42	11	16
Female	12.88	49	39	8	14
Male	13.52	51	60	7	16
Male	13.67	58	44	11	11
Female	13.70	64	58	10	15
Female	13.78	58	40	10	17
Male	13.89	57	46	18	13
Male	14.38	62	56	11	18
Female	14.52	59	50	10	19
Male	15.11	49	42		
Male	15.45	63	60	15	16
Male	15.65	55	48	11	20
Female	15.67	40	50	9	20
Male	15.68	60	39	10	21
Female	15.81	62	60	7	20
Male	16.03	55	51	11	21
Male	16.27	51	58	11	23
Male	16.30	56	62	13	19
Female	16.33	51	47	7	19
Male	16.54	49	38	12	17
Female	16.65	55	56	17	21
Male	16.77	52	52	5	15
Female	16.84	42	46	11	14
Male	17.07	55	58	18	18
Female	17.26	48	47	7	18
Female	17.27	49	48	5	15
Female	17.33	49	42	11	22
Female	17.41	53	59	11	17
Female	17.41	63	57	8	34
Male	17.52	51	35	14	21
Male	17.73	30	30	8	14
Male	18.56	48	51	10	21
MIN	12.38	30.00	30.00	5.00	11.00
MAX	18.56	64.00	62.00	18.00	34.00
AVG	15.69	52.79	48.88	10.47	18.25
STDEV	1.67	7.23	8.41	3.29	4.10
MIN-Z	−1.98	−3.15	−2.24	−1.66	−1.77
MAX-Z	1.72	1.55	1.56	2.29	3.85
MIN-ZC	−1.98	−2.24	−1.82	−1.69	−2.34
MAX-ZC	1.72	1.83	1.63	2.21	1.75

### Psychological questionnaires

#### Attachment style

Participants completed a validated French version of the Relationships Scales Questionnaire (RSQ; Griffin and Bartholomew 1994; Guedeney et al. 2010). The RSQ was analyzed according to recent recommendations by Kurdek (Kurdek 2002), yielding “… *psychometrically sound scores of attachment styles* …” (p. 831), particularly in comparison with the attachment interview. The avoidance dimension was calculated by summing up eight items, and the anxiety dimension by adding up five of the total 30 items, and centered Z-scores were used in a bidimensional manner for further analysis (Vrticka et al. 2008, 2012a,b).

### Experimental task

Our social feedback paradigm consists of a difficult perceptual game during which participants have to rapidly count and compare the number of dots appearing in two dot clouds to the left and right of a centered line (see Fig. 1). After each trial, feedback is provided to participants, consisting of a word (either “Won” or “Lost”) and a face (either with a smiling or an angry emotional expression). The word always indicates the participants' objective performance during the preceding trial—correct responses are consistently paired with the word “Won”, and incorrect responses with the word “Lost”. The emotional facial expression, however, does not follow such a predetermined rule. A smiling expression can be paired with both “Won” and “Lost” words, the same applying for an angry expression. Through such experimental manipulation, two congruent (smiling face won—SFW: *social support*—and angry face lost—AFL: *social punishment*), and two incongruent (smiling face lost—SFL: *schadenfreude/gloating*—and angry face won—AFW: *resentment*) word-face pairings are created. This manipulation readily induces the perception of “friends” (congruent feedback) versus “foes” (incongruent feedback), because, for example, a smiling expression perceived after a successful trial (“Won”) has a very different social implication than a smiling expression seen after an unsuccessful trial (“Lost”; Vrticka et al. 2008). The task therefore delineates four well-differentiated and predefined congruent versus incongruent social evaluations of one's own objectively evaluated performance (see above and Methods, as well as Vrticka et al. 2008). In terms of attachment style, the chosen experimental paradigm appears particularly well suited to reveal individual differences in the processing of social feedback, because previous studies report prominent modulation of brain activity to faces as a function of both attachment avoidance and anxiety (see e.g., Strathearn et al. 2009; Suslow et al. 2009; Donges et al. 2012).

The total number of dots and the difference between the two display sides were adjusted online based on the participant's performance on preceding trials, by reducing the difference after each correct trial (minimum one dot) or increasing the difference after each incorrect trial (maximum five dots), allowing us to maintain performance close to threshold and to obtain approximately equal numbers of correct and incorrect trials. In addition, to further ensure this equal distribution, occasional displays with 15 dots on both sides were inserted whenever performance exceeded 60% correct of two consecutive trials. None of the participants noticed these “trick” trials (Vrticka et al. 2008).

Participants were instructed about the paradigm according to a written transcript to ensure that everybody understood the aim of the paradigm in the same way. Participants were told that they would be involved in a difficult visual task and that we would measure their performance—the aim therefore was to give as many correct responses as possible as fast as possible. Participants then were instructed that after each trial, there would be a feedback consisting of a word and a face. The word would give them feedback about their performance, and the face would additionally provide them with emotional evaluations from people either being “friendly” or “unfriendly” (see also Vrticka et al. 2008).

### Data acquisition and imaging

Scanning was performed at the Brain and Behavior Laboratory (BBL), University of Geneva Medical Center, on a 3T Trio MRI scanner manufactured by Siemens (Erlangen, Germany). Standard functional EPI T2*-weighted volumes were collected as thirty eight 3.2 mm contiguous axial slices: TR = 2.4 s, TE = 30 ms, Flip Angle = 85°, FOV: 235 mm, in-plane resolution of 2.4 by 1.8 mm. One structural T1-weighted image using standard MPRAGE sequence was also collected from each subject (TR = 2.5 s, TE = 3 ms, TI = 1.1 s, voxel size 1.1 mm^3^). Presentation of the stimuli and collection of the behavioral responses from the subjects were made using E-Prime software Version 2.0, RRID: nlx_155747 (PST Software Inc., Pittsburgh, PA).

### Data analysis

Questionnaire and demographic data were analyzed using Excel 2007 (Microsoft, Redmond, PA) and SPSS (http://www-01.ibm.com/software/analytics/spss/), including assessment on their distribution and mean scores, and values were centered (Z-scores) to avoid collinearity issues (Aiken and West 1991). Relations between behavioral data and questionnaire measures as well as age were computed using multiple regression analyses, by entering performance as dependent, and attachment questionnaire scores as well as age and sex as independent variables. A separate independent samples *t*-test analysis was computed regarding sex differences by controlling for attachment avoidance, anxiety, and age.

Functional images were analyzed using SPM Version 8, Revision Number 4290, RRID: nif-0000-00343 (Wellcome Department of Imaging Neuroscience, London, UK; http://www.fil.ion.ucl.ac.uk/spm) under Matlab. EPI volumes were realigned, normalized to the MNI (Montreal Neurological Institute) template, resampled to 2 mm^3^, and spatially smoothed using a 8-mm FWHM Gaussian kernel. Coordinates thus refer to millimeters in the MNI stereotaxic space.

For each participant, the six different conditions (SFW, SFL, AFW, AFL, as well as neural activity during dot perception, either on “Won” [DW] or “Lost” [DL] trials—used as a baseline) were modeled as single events and convolved with the standard hemodynamic response (Vrticka et al. 2008). The first-level model also included four additional conditions (SFW-M, SFL-M, AFW-M, and AFL-M) representing brain activity during a subsequent memory task, because this memory task was scanned immediately after the session of interest for the present investigation. However, these additional memory conditions will not be considered here. Realignment parameters were incorporated as six additional regressors of no interest. During the estimation of the model, a high-pass frequency filter (cutoff 128 s) and corrections for autocorrelation between scans were applied to the time series. Random effects were evaluated by combining contrast images computed from individual analyses.

First, we computed the main effects contrast of objective performance feedback ([SFW + AFW] vs. [SFL + AFL]), facial emotional expressions ([SFW + SFL] vs. [AFW + AFL]), and their interaction (congruent [SFW + AFL] vs. incongruent [SFL + AFW] social evaluation), and further decomposed these contrasts by computing comparisons between two experimental conditions only (e.g., SFW vs. AFW, etc.). These analyses were carried out at *P* < 0.001 uncorrected at the peak and *P* < 0.05 FWE corrected at the cluster level, with a voxel extent of *k* = 20. This served to assess brain activity underlying social feedback processing for the whole adolescent participant sample in general.

Subsequently, we derived a two-sample *t*-test for the congruent versus incongruent contrast to examine any sex differences during the processing of social feedback, by simultaneously controlling for attachment avoidance, anxiety, and age. Statistical threshold for this analysis was set at *P* < 0.001 uncorrected and *k* = 20 (Lieberman and Cunningham 2009).

Finally, we computed a whole-brain multiple regression analysis (wbMRA) for the contrast congruent versus incongruent, again at *P* < 0.001 uncorrected and *k* = 20. Covariates were age, attachment avoidance, and anxiety, as well as sex. Continuous variables were centered (Z-scores) to avoid collinearity issues (Aiken and West 1991). This wbMRA approach ensured that effects observed for one particular covariate were controlled for the influence of all other covariates.

To decompose and illustrate the found associations in regions of interest (sex differences, interactions among age, attachment avoidance and anxiety, and brain activation difference for congruent versus incongruent social feedback trials), raw activity (betas) was extracted and further processed using SPSS. This included the production of partial regression plots to depict the significant effects as observed by means of SPM analyses. Furthermore, separate assessment of sex differences and correlations between age, attachment avoidance or anxiety and brain activity for congruent and incongruent social feedback trials and their decomposition into the four separate experimental conditions were derived by the means of additional multiple regression analyses on extracted betas using SPSS, and again illustrated by partial regression plots.

## Results

### Psychological questionnaires and demographic data

Test of continuous variable distributions (“outlier” analysis) revealed that one participant scored too low on externalizing (Z-Score = −3.15; exclusion criterion), and one participant scored too high on attachment avoidance (Z-Score = 3.85; outlier) scores (see Table 1). These two participants were therefore removed from any further correlational analyses. One additional participant had missing attachment scores and was therefore also removed from all further correlational analyses. The remaining *N* = 30 participants were included in the correlational analyses.

Analysis of questionnaire data as well as age for these *N* = 30 participants revealed no significant associations between attachment and maladaptive functioning, and attachment avoidance and anxiety (*P* < 0.05). In turn, there was a positive association between age and attachment avoidance (Pearson-*r* = 0.438, *P* = 0.015).

For age and attachment measures (*N* = 12 female), no sex differences were found (*P* > 0.05).

### fMRI data

**Figure 2:**
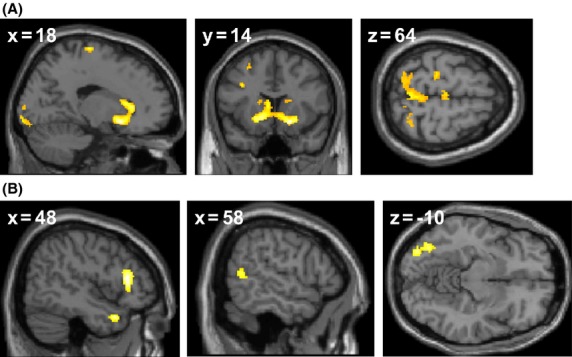
Brain areas activated by (A) the main effects contrast of performance feedback (WON > LOST), and (B) social feedback processing (INCONGRUENT > CONGRUENT). For illustration purposes, statistical threshold is set at *P* < 0.001 uncorrected and *k* = 20. Activation maps are overlaid on a single subject anatomical T1 template.

We first computed all main effects contrasts of interest, including (1) main effect of objective performance feedback (won vs. lost), (2) main effect of emotional facial expression (smile vs. angry), and (3) the performance × emotion interaction (social feedback; contrast congruent vs. incongruent), as well as their further decompositions. This was done in *N* = 33 participants (*N* = 14 female). Results are summarized in Table 2. Increased activity was observed for the main effect of objective performance feedback (won > lost) and its respective decompositions (i.e., SFW > SFL and AFW > AFL) in areas including the bilateral ventral striatum, caudate, nucleus accumbens, medial prefrontal cortex, and right pre/postcentral gyrus (see Fig. 2A). The objective performance feedback × emotional facial expression interaction (social feedback; contrast incongruent > congruent) revealed heightened blood-oxygenation-level-dependent (BOLD) signal change in right inferior frontal gyrus and left temporo-occipital cortex, in addition to the right temporo-occipito-parietal junction for its decomposition (contrast AFW > SFW; see Fig. 2B).

**Table 2:** Regions activated by the main effects contrasts of objective performance feedback, emotional facial expression, and social feedback (congruent vs. incongruent interaction). Coordinates are given in MNI space. BA, Brodmann area. A statistical threshold of *P* < 0.001 uncorrected at the peak and *P* < 0.05 FWE-corrected at the cluster level applies for all regions

Won versus Lost				
Voxel	*P*-value Cluster (FWE-cor. *P* < 0.05)	*x, y, z*	Region	BA
1146	<0.001	18, 14, −10	Ventral striatum/caudate/nucleus accumbens bilateral	
1749	<0.001	4, −44, 70	Postcentral gyrus right	BA 3/4/5/6/7
298	0.004	34, −66, 50	Superior parietal lobule	BA 7/40

Lost versus Won				
—				

Smile versus Angry				
—				

Angry versus Smile				
—				

Congruent versus Incongruent				
—				

Incongruent versus Congruent				
Voxel	*P*-value Cluster (FWE-cor. *P* < 0.05)	*x, y, z*	Region	BA
263	0.008	46, 26, 14	Inferior frontal gyrus right	BA 45
216	0.018	−30, −84, −8	Inferior occipital gyrus left	BA 18/19

SFW versus AFW				
—				

AFW versus SFW				
Voxel	*P*-value Cluster (FWE-cor. *P* < 0.05)	*x, y, z*	Region	BA
274	0.006	48, −52, 2	Temporo-occipital-parietal-junction right	BA 22/39

SFW versus SFL				
Voxel	*P*-value Cluster (FWE-cor. *P* < 0.05)	*x, y, z*	Region	BA
2866	<0.001	−20, −28, 66	Superior parietal lobule/precentral gyrus/supplementary motor area/ventral striatum/caudate/nucleus accumbens left	
531	<0.001	14, 18, −10	Ventral striatum/caudate/nucleus accumbens right	

SFL versus SFW				
—				

SFL versus AFL				
—				

AFW versus AFL				
Voxel	*P*-value Cluster (FWE-cor. *P* < 0.05)	*x, y, z*	Region	BA
229	0.014	14, 6, −6	Ventral striatum/caudate/nucleus accumbens right	
279	0.006	−14, 12, −4	Ventral striatum/caudate/nucleus accumbens left	
238	0.012	−20, −96, −10	Lingual gyrus left	BA 17/18

Subsequently, we tested for the influence of participant sex, age, and attachment style measures on the perception of social feedback (objective performance feedback × emotional facial expression interaction; contrast incongruent vs. congruent) specifically. This was done in *N* = 30 participants (*N* = 12 female). Findings are summarized in Table 3.

**Table 3:** Regions activated by the whole-brain multiple regression analysis (contrast congruent vs. incongruent) regarding age and attachment avoidance and anxiety, as well as the two-sample *t*-test regarding sex differences. Coordinates are given in MNI space. BA, Brodmann area. A statistical threshold of *P* < 0.001 uncorrected at the peak applies for all clusters

Congruent versus Incongruent × Avoidance Positive				
Voxel	*Z*-value	*x, y, z*	Region	BA
261	4.69	10, 14, 18	Caudate right	
320	4.55	−34, −2, 2	Hippocampus left	
133	4.38	−16, −12, −14	Amygdala/Hippocampus left	
76	4.36	−34, −66, −2	Middle occipital gyrus left	
71	4.18	−14, −14, 30	Cingulate gyrus left	
69	4	4, 38, 0	Ventral anterior cingulate cortex	BA 24/32
56	3.96	36, 12, −10	Anterior insula/Inferior frontal gyrus right	BA 47
109	3.94	30, −34, 0	Hippocampus right	
39	3.72	4, −4, 26	Cingulate gyrus	BA 24
35	3.48	−14, 8, 28	Cingulate gyrus	
22	3.48	10, −24, −6	Midbrain	

Congruent versus Incongruent × Anxiety Negative				
Voxel	*Z*-value	*x, y, z*	Region	BA
999	4.69	10, −50, −36	Cerebellum	
143	4.17	34, 28, 6	Anterior insula/inferior frontal gyrus right	BA 7/45
31	4.11	−36, −66, 0	Middle occipital gyrus left	
42	3.79	32, −76, 2	Middle occipital gyrus right	
23	3.77	−36, 44, −10	Middle frontal gyrus left	BA 11

Congruent versus Incongruent × Age Negative				
Voxel	*Z*-value	*x*, *y*, *z*	Region	BA
34	4.18	0, 36, −4	Ventral anterior cingulate cortex	BA 32
36	3.58	36, 14, −10	Anterior insula/inferior frontal gyrus right	BA 13

Congruent versus Incongruent × Sex				
Voxel	*Z*-value	*x*, *y*, *z*	Region	BA
50	4.09	24, 44, 22	Superior frontal gyrus right	BA 10
51	4.06	−10, 60, 22	Medial frontal gyrus left	BA 9/10
33	3.81	28, 20, 32	Middle frontal gyrus right	
20	3.63	10, 14, 18	Caudate right	

**Figure 3:**
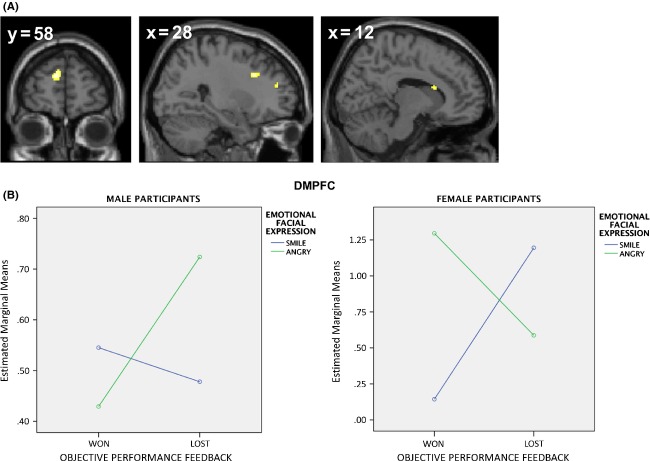
Brain areas within which there were significant sex differences during social feedback processing (contrast CONGRUENT vs. INCONGRUENT). (A) Activation maps overlaid on a single subject anatomical T1 template. Statistical threshold is set at *P* < 0.001 uncorrected and *k* = 20. (B) Plotted raw-activation values (betas) extracted, averaged, and decomposed into the four experimental conditions from the dorsomedial prefrontal cortex (DMPFC). BOLD signal change is shown on the left for male participants, and on the right for female participants. Male participants showed a congruent > incongruent effect, whereas female participants showed an incongruent > congruent effect. A very similar activation pattern was present in the remaining three brain areas displaying sex differences.

Significant sex differences in the processing of social feedback were observed in bilateral middle, medial, and superior frontal gyrus (overlapping with the dorsomedial prefrontal cortex—DMPFC—and [dorso]lateral prefrontal cortex—[D]LPFC), as well as in the right caudate (see Fig. 3A, B). In all of these four brain areas, females had higher differential activity to incongruent but males to congruent social feedback.

**Figure 4:**
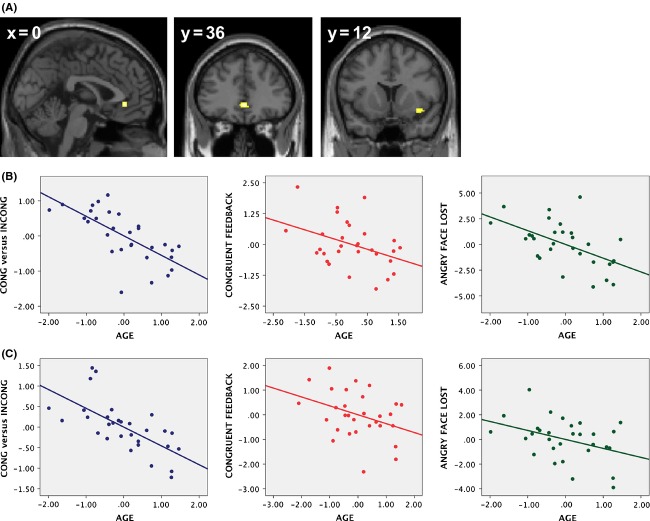
Brain areas within which there were significant correlations with age during social feedback processing. (A) Activation maps overlaid on a single subject anatomical T1 template at a statistical threshold of *P* < 0.001 uncorrected and *k* = 20, depicting the anterior cingulate cortex (left and middle) as well as anterior insula (right). Partial regression plots depicting associations between age and brain activity during social feedback processing in the anterior cingulate cortex (B) and anterior insula (C) are shown below. Left panel: BOLD signal representing a CONGRUENT versus INCONGRUENT ratio (*y*-axis) is plotted against age (*x*-axis, centered values) in a dimensional manner. Middle panel: BOLD signal representing CONGRUENT feedback only (*y*-axis) is plotted against age (*x*-axis, centered values) in a dimensional manner. Right panel: BOLD signal representing ANGRY FACE LOST feedback only (*y*-axis) is plotted against age (*x*-axis, centered values). Brain activity reflects averaged values across all significant voxels of activated clusters.

Increasing age was negatively associated with differential brain activity to incongruent versus congruent social feedback in ventral anterior cingulate cortex (vACC) and right anterior insula/inferior frontal gyrus (aINS; see Fig. 4A, B, C). Decomposition of these findings revealed that such shift in stronger processing from congruent (younger adolescents) to incongruent (older adolescents) social feedback with age appeared to be mainly driven by a decrease in BOLD signal change to congruent social feedback (multiple regression analysis; vACC: *P* < 0.05; aINS: *P* = 0.064), and particularly the AFL condition (vACC: *P* < 0.01; aINS: *P* < 0.05).

**Figure 5:**
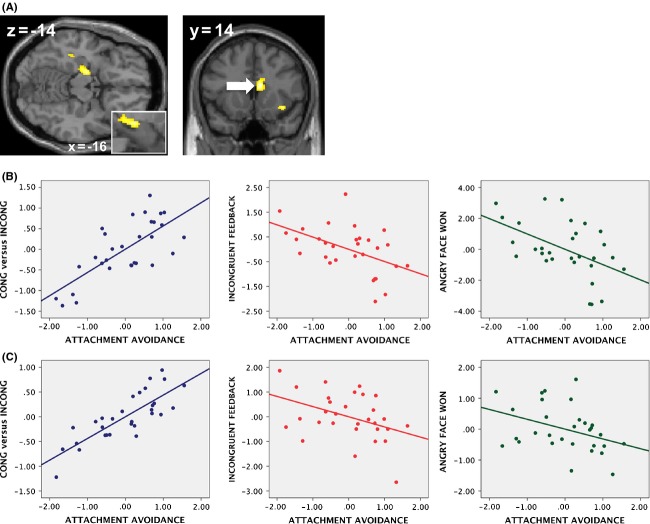
Significant correlations with attachment avoidance during social feedback processing in the amygdala/hippocampus and caudate. (A) Activation maps overlaid on a single subject anatomical T1 template at a statistical threshold of *P* < 0.001 uncorrected and *k* = 20, depicting the anterior cingulate cortex (left), and caudate (white arrow, right). Partial regression plots depicting associations between attachment avoidance and brain activity during social feedback processing in the amygdala (B) and caudate (C) are shown below. Left panel: BOLD signal representing a CONGRUENT versus INCONGRUENT ratio (*y*-axis) is plotted against attachment avoidance (*x*-axis, centered values) in a dimensional manner. Middle panel: BOLD signal representing INCONGRUENT feedback only (*y*-axis) is plotted against attachment avoidance (x-axis, centered values) in a dimensional manner. Right panel: BOLD signal representing ANGRY FACE WON feedback only (*y*-axis) is plotted against attachment avoidance (*x*-axis, centered values) in a dimensional manner. Brain activity reflects averaged values across all significant voxels of activated clusters.

**Figure 6:**
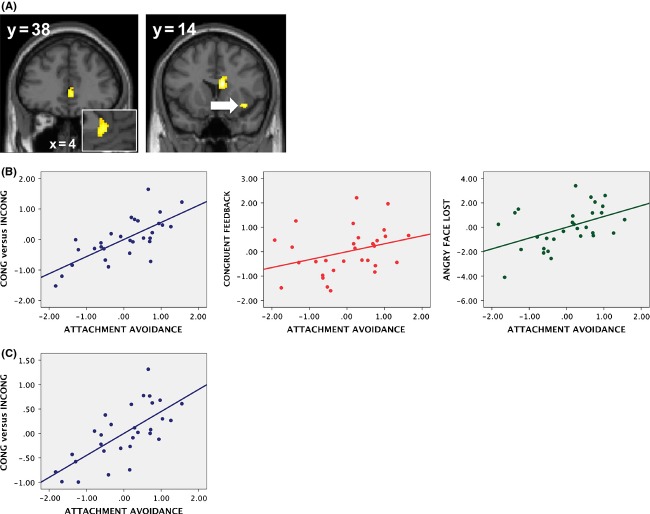
Significant correlations with attachment avoidance during social feedback processing in the ventral anterior cingulate cortex and anterior insula. (A) Activation maps overlaid on a single subject anatomical T1 template at a statistical threshold of *P* < 0.001 uncorrected and *k* = 20, depicting the anterior cingulate cortex (left), and anterior insula (white arrow, right). Partial regression plots depicting associations between attachment avoidance and brain activity during social feedback processing in the amygdala (B) and caudate (C) are shown below. Left panel (both (B) and (C)): BOLD signal representing a CONGRUENT versus INCONGRUENT ratio (*y*-axis) is plotted against attachment avoidance (*x*-axis, centered values) in a dimensional manner. Middle panel ((B) only): BOLD signal representing CONGRUENT feedback only (*y*-axis) is plotted against attachment avoidance (*x*-axis, centered values) in a dimensional manner. Right panel ((B) only): BOLD signal representing ANGRY FACE LOST feedback only (*y*-axis) is plotted against attachment avoidance (*x*-axis, centered values) in a dimensional manner. Brain activity reflects averaged values across all significant voxels of activated clusters.

Attachment avoidance was positively associated with differential brain activity to congruent versus incongruent social feedback in the vACC, left amygdala/hippocampus and bilateral posterior hippocampus, right anterior insula/inferior frontal gyrus (aINS), right caudate, and left middle occipital gyrus (see Fig. 5A, B, C and Fig. 6A, B, C). In other words, in the above regions, increasing attachment avoidance scores were linked with a shift in processing from incongruent to congruent social feedback. The putative underlying mechanisms of this shift, however, differed considerably. In the amygdala/hippocampus and caudate, attachment avoidance appeared to be particularly associated with a decrease in activity during incongruent social feedback processing (multiple regression analysis; amygdala/hippocampus: *P* < 0.01; caudate: *P* < 0.05), affecting both the AFW and SFL conditions (amygdala/hippocampus: *P* < 0.05; caudate; *P* = 0.052 and *P* = 0.073). Instead, in the vACC and aINS, attachment avoidance was linked with an increase of BOLD signal change to congruent social feedback, although this effect did not reach significance (multiple regression analysis; vACC: *P* = 0.098; aINS: *P* = 0.16), and mainly driven by increased BOLD signal change to the AFL condition (vACC: *P* < 0.05; aINS: *P* = 0.099).

**Figure 7:**
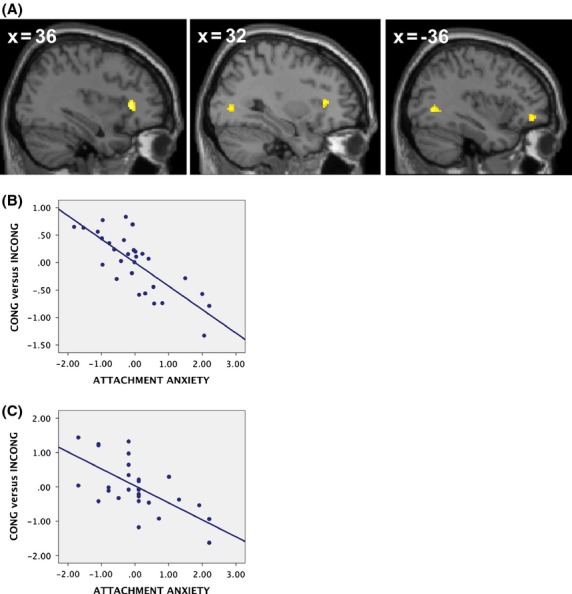
Brain areas within which there were significant correlations with attachment anxiety during social feedback processing. (A) Activation maps overlaid on a single subject anatomical T1 template at a statistical threshold of *P* < 0.001 uncorrected and *k* = 20, depicting the anterior insula (left and middle), occipital gyrus (middle and right), as well as ventrolateral prefrontal cortex (right). Partial regression plots depicting associations between attachment anxiety and brain activity during social feedback processing in the anterior insula (B) and ventrolateral prefrontal cortex (C) are shown below. BOLD signal representing a CONGRUENT versus INCONGRUENT ratio (*y*-axis) is plotted against attachment anxiety (*x*-axis, centered values) in a dimensional manner. Brain activity reflects averaged values across all significant voxels of activated clusters.

Finally, increasing attachment anxiety was negatively associated with differential brain activity to congruent versus incongruent social feedback in aINS, left middle frontal gyrus (ventrolateral prefrontal cortex – VLPFC), bilateral middle occipital gyrus, and cerebellum (see Fig. 7A, B). This activation pattern suggests that increasing anxious attachment scores were associated with a shift in processing from congruent to incongruent social feedback. Further decomposition did, however, not reveal any significant simple associations between attachment anxiety and BOLD signal change to congruent or incongruent social feedback, or the four specific experimental conditions.

## Discussion

This fMRI study aimed at investigating the underlying neural activation of social feedback processing during adolescence. While examining general activation patterns across our entire sample of 33 adolescents aged from 12 to 19, we were particularly interested in probing for possible differences in cerebral activity when comparing congruent versus incongruent social feedback activations, and assessing the potential influences of sex, age, and attachment style on these neural patterns. We found that social feedback processing in our adolescent sample was most observable in terms of brain activation during incongruent (versus congruent) social feedback conditions. These results are consistent with previous findings obtained in a sample of healthy adults (Vrticka et al. 2008). In addition to these main effects of social congruency on brain activation patterns, our analyses further revealed effects of age, sex, and attachment style on neural activation during social feedback processing. Whereas we observed increased brain activation during incongruent social feedback processing in females, older participants, and individuals with an anxious attachment style, we found stronger congruent social feedback processing activity in males and participants with an avoidant attachment style. The distinct underlying mechanisms and their implications in terms of adolescent development as well as attachment theory are discussed in more detail below.

### Social feedback processing in adolescents

The paradigm employed in this study combined a written performance evaluation (either “won” or “lost”) with an emotional facial display (either smiling or angry face), which together composed to social feedback stimuli processed by adolescent participants. Consistent with our predictions, contrasting “won” to “lost” trials revealed reward-related brain responses including the ventral striatum (putamen, caudate, nucleus accumbens) (Vrticka et al. 2008; Sescousse et al. 2013). In addition, when analyzing the interaction effects (performance feedback × social evaluation) or in other words congruency effects of the social feedback stimuli, the most pervasive effects were observed for the contrasts incongruent > congruent for angry over smiling faces. Activations included the inferior frontal gyrus (IFG) and temporo-occipital-parietal junction (TOPJ). This set of *in*congruity effects suggests the potential involvement of multiple brain mechanisms when processing incongruent social feedback. Indeed, while the IFG has often been associated with emotion regulation processes (Payer et al. 2012), the TOPJ has often been linked to theory of mind and incongruity detection (Vrticka et al. 2013). More generally, the significant interaction effects are consistent with the notion that the adolescent brain processes emotional facial expressions in an integrative manner by taking into account behavioral outcomes (game performance), rather than like fixed templates as suggested by basic emotion theory (see Vrticka et al. 2008). Further investigations allowing for direct comparisons between adolescents and adults, and the inclusion of younger participants to test for early developmental effects—ideally in a longitudinal manner—are motivated by these preliminary results.

### Sex differences in social feedback processing during adolescence

Across the entire sample of adolescent participants (controlled for age and attachment style), we observed differentially increased activity in the middle, medial, and superior frontal gyrus as well as the caudate during incongruent social feedback processing in females, but congruent feedback processing in males.

The above-mentioned middle frontal gyrus cluster overlaps with an area of the dorsomedial prefrontal cortex (DMPFC). This DMPFC region has been found involved in both self- and other-reflection by a recent quantitative meta-analysis of social cognitive studies (participants aged 17–41 years; Murray et al. 2012). More specifically, in this meta-analysis, the DMPFC was attributed to a particular role in inferential evaluation of externally generated social information. This inferential evaluation is argued to serve the aim of understanding the plans and motives of others in relation to the self, and to adapt one's own feelings and behavior accordingly (Murray et al. 2012). Such interpretation of DMPFC activity is corroborated by another recent meta-analysis, which found the DMPFC to be prominently associated with perspective-taking and metacognition-related processing in social cognition (Bzdok et al. 2013). Applied to this study, differential DMPFC activity during the processing of incongruent versus congruent social feedback as a function of participant sex could represent additional cognitive effort to monitor and/or update one's self-representation in the face of either incongruent (female) or congruent (male) social information.

A similar activation pattern was also observed in the medial and superior frontal gyri (overlapping with the [Dorso]lateral prefrontal cortex; [D]LPFC), as well as caudate. In previous social cognitive neuroscience studies specifically involving self- and other-reflection the superior frontal gyrus has been linked with processes related to emotional introspection and self-judgment, also involving psychological distancing—the ability to view social stimuli as an affectively detached and objective observer (Murray et al. 2012). Such processes overlap with more general functions of the superior frontal gyrus including the DLPFC in top-down modulation and cognitive control (Miller and Cohen 2001). In turn, increased BOLD signal change in the caudate has been associated with the attribution of self-relevance or self-relatedness—in this context being defined as the valuing of external and internal stimuli with regard to their meaning for the organism (see Enzi et al. 2009). In our study, the observed differential activity for congruent (male) versus incongruent (female) social feedback could therefore suggest a distinct predisposition of adolescents to search for clues in their social environment either corresponding, or being opposed to, objective performance feedback.

Overall, our findings regarding sex differences in relation to congruency during social feedback processing provide preliminary experimental data suggesting that, as far as localization of cerebral activation is concerned, our female adolescent group engaged to a greater degree in incongruent social feedback processing as compared to our male adolescent group. This pattern may be related to the general impression that females are more prosocially oriented, to the extent that they would more willingly consider discrepant social feedback in the way they monitor and/or update their self-representation in a social context.

### Age effects on social feedback processing during adolescence

Examining the effects of age (controlled for attachment style and sex), we found evidence for increased differential activity to incongruent social feedback in the ventral anterior cingulate cortex (vACC) and anterior insula (aINS) in older adolescents. In both brain areas, this pattern emerged because activity decreased for congruent social feedback, and particularly the AFL condition, as a function of participant age.

The vACC is generally thought to be implicated in emotional regulation as well as socio-emotional evaluation and conflict resolution (Somerville et al. 2006; Lieberman 2007; Kanske and Kotz 2011). Previous data from adults furthermore suggest that conflict resolution more strongly activates the vACC if processed information is perceived as (self-)relevant (Moran et al. 2006; Kanske and Kotz 2011). Building on such previous accounts, our findings may indicate that the development of social feedback processing during adolescence could be characterized by a decreasing focus of self-relevance when regarding congruent social feedback, and particularly negative social feedback after an objective failure (AFL condition).

Similar to results in the vACC, activity in the aINS for congruent trials, and particularly the AFL condition, showed a decrease with age. Because the aINS is thought to sustain an internal, visceral, and embodied representation of social information processing (Gallese 2010; Murray et al. 2012), the present results would motivate the application of appropriate paradigms to investigate the link between social information processing and visceral reactions with a sample of adolescents. On the basis of the current paradigm, a possible working hypothesis would propose an association between visceral responses at the time of incongruent social feedback.

### Attachment effects on social feedback processing during adolescence

Our investigation also aimed at examining the association between attachment insecurity and social feedback processing during adolescence. While we observed attachment anxiety to mirror above-described age effects on congruent (versus incongruent) social feedback processing, attachment avoidance was marked by an apparent inversion of such associations.

Our data revealed that increasing scores on the anxious attachment dimension were marked by a shift in processing from congruent to incongruent social feedback in the aINS and middle frontal gyrus (overlapping with the ventrolateral prefrontal cortex [VLPFC]). According to the above discussion of aINS involvement in the embodiment of intersubjectivity (Murray et al. 2012), our findings regarding attachment anxiety may suggest that highly anxiously attached adolescents were more strongly viscerally representing emotional conflict induced by incongruent social feedback. Similar patterns were observed for VLPFC activations. The VLPFC is typically associated with emotion regulation processes (Lieberman 2007), also including social relationship contexts (Koban et al. 2014). Along these lines, the VLPFC may mediate the regulation and resolution of social conflicts involving the representation of similar versus dissimilar self- and other-representations. Applied to this study, increased VLPFC activity to incongruent social feedback in high anxiously attached individuals suggests stronger involvement of emotion regulation processes. Because we did not find any significant associations between attachment anxiety and congruent and incongruent social feedback processing as well as the four specific experimental conditions in the aINS and VLPFC *per se*, we cannot further specify the observed activation patterns. Targeted research is needed to address these outstanding questions.

To the extent that neuroimaging results can speak to conceptual work originating from attachment theory, it has been hypothesized that attachment anxiety is characterized by an increase in the activation of the attachment system, particularly under conditions of social stress or conflict (Mikulincer and Shaver 2007; Vrticka and Vuilleumier 2012). Anxiously attached individuals are described as especially sensitive to social clues of rejection and/or punishment. In the adult neuroimaging literature, some supporting evidence comes from the relationship between attachment anxiety and increased brain activity, including the amygdala and aINS (Vrticka et al. 2008, 2012a; DeWall et al. 2012). The present data lend further support by observing an increasing focus on social information representing a potential interpersonal conflict (incongruent trials). Importantly, the current report suggests that such associations can be observed during adolescence.

Of note is the fact that our findings regarding attachment anxiety and age show a similar overall activation pattern in the aINS. In addition, the observed global association between attachment anxiety and brain activity in the VLPFC to incongruent social feedback also points into the same direction. It therefore appears that an anxious attachment style, as measured here in a sample of healthy controls, may be, to a certain degree, consistent with maturational age effects. In fact, developing concern for the opinion of others, in moderation, may prove to be adaptive in social interactions. However, we may ask at which level (threshold) such influence of anxious attachment on concern for social evaluation becomes maladaptive or even too disruptive for interpersonal processes. The present design is insufficient to address such issues, and studies including clinical populations with severe attachment anxiety are needed.

In contrast to attachment anxiety, which was found to mirror age-dependent effects during incongruent social feedback processing, attachment avoidance appears to have influenced social feedback processing in the opposite way. We observed attachment avoidance to be associated with a shift in processing from incongruent to congruent social feedback in the amygdala/hippocampus, caudate, vACC, and aINS. The underlying mechanisms, however, differed considerably.

On one hand, in the amygdala/hippocampus and caudate, we found that this shift in processing from incongruent to congruent social feedback was mainly due to a decrease in BOLD signal change to incongruent social feedback, affecting both the AFW and SFL conditions. The human amygdala is nowadays typically understood to function as a relevance detector, responding more strongly to information that is relevant to the intentions and goals of a given person at a particular moment in time (Sander et al. 2003; Pessoa and Adolphs 2010). Such relevance processing is then likely to affect memory-related mechanisms maintained by the hippocampus (see e.g., Vrticka et al. 2009). On a similar note, the caudate has been previously associated with the encoding of self-relevance/ self-relatedness, associated with the valuing of external and internal stimuli with regard to their meaning for the organism (see Enzi et al. 2009). From these data, it appears that in the present experiment, increasing attachment avoidance may have entailed a decrease in the attribution of self-relevance to incongruent social feedback.

On the other hand, we observed attachment avoidance to be associated with a shift in the processing from incongruent to congruent social feedback in the vACC and aINS - likely characterized by increased responses to congruent social feedback, and particularly the AFL condition. Such stronger emotional and visceral reactions to congruent social information with increasing avoidance, especially in the case of negative social feedback after an objective failure, may inform on the type of internal/subjective experience that are likely triggered in avoidantly attached adolescents during social feedback processing.

Notwithstanding the limitations of interpreting neuroimaging results in reference to conceptual work originating from attachment theory, attachment avoidance has been described by a general downregulation of the attachment system. Such downregulation is argued to represent a (secondary attachment) strategy to prevent the experience and/or expression of strong emotions in social contexts (Mikulincer and Shaver 2007; DeWall et al. 2012; Vrticka and Vuilleumier 2012; Vrticka et al. 2012a). Our data suggest that one strategy avoidantly attached individuals may use to maintain their attachment system in a low activation state is to attribute less self-relevance to conflicting social information (i.e., incongruent social feedback), and to instead more readily process confirmatory social feedback, also on the emotional and visceral levels.

In contrast with adult data showing decreased behavioral and neural responding to congruent, and particularly positive social feedback (Vrticka et al. 2008, 2012b; Strathearn et al. 2009), attachment avoidance during adolescence appears to be associated with somewhat different neural mechanisms. One potential explanation for such discrepancies may be the fact that during adolescence, reactions to socio-emotional information established within the family context are challenged by interactions with peers and unknown others, which in turn might offer the possibility for change and adaptation. More research is, however, clearly needed in the future to explore this hypothesis.

The above said, what stands out regarding attachment avoidance is that brain activation patterns appear to go against observed age effects in the overall adolescent sample. Our results suggest that older age is likely associated with an increase in social sensitivity—reflected by heightened social conflict resolution and associated embodied representations of intersubjectivity during incongruent social feedback processing. Such mechanism, in turn, is probably linked with decreased relevance attribution to congruent social feedback. Conversely, we observed that high attachment avoidance may be linked with decreased self-relevance/ self-relatedness processing of incongruent social feedback, and increased orientation of emotional conflict resolution mechanisms toward congruent social feedback. High attachment avoidance may therefore preclude the usually observed “opening up” to social information in terms of social sensitivity, here reflected by weaker brain responses to incongruent social feedback. Opposed to the age-consistent effects of attachment anxiety on social feedback processing, attachment avoidance may incur less mature processing of social feedback. It is of great interest to confirm and further extend such findings in future investigations also including clinical participants with severe attachment avoidance.

Finally, we note that we did not observe any associations between social feedback processing and attachment avoidance in the somatosensory cortex as reported previously during masked presentation of sad faces (Suslow et al. 2009). Such differences are likely due to the fact that our four stimulus conditions did not comprise sadness, and our paradigm relied on longer stimulus exposure as well as explicit task parameters.

## Limitations

One potential limitation of the present investigation could be seen in the fact that we measured adolescents' developmental state by means of age and not pubertal status (van den Bos 2013). We can therefore not disentangle age- and puberty-related developmental changes. This being said, it is still not clear how exactly hormonal effects differ from age-related influences on socio-emotional brain processes, and how these two variables potentially interact with each other. Future studies comparing the use of age as a categorical versus dimensional (as applied here) measure, including pubertal status as an additional variable, are encouraged.

Another possible limitation is the fact that the participant population of this study only included adolescents. In order to deduce developmental trajectories of the transition between adolescence and adulthood, future studies with a cross-sectional or longitudinal design are required.

## Conclusions

The present fMRI investigation provides preliminary evidence for the neural correlates of adolescents' social feedback processing, in association with significant dimensions such as sex, age, as well as attachment style. We found indication for increased social sensitivity in terms of heightened incongruent social feedback processing in females, older participants, and individuals with an anxious attachment style. In turn, we observed a stronger tendency for avoiding social conflict and associated social adaptation through preferential processing of congruent social feedback in males and participants with an avoidant attachment style. Future studies are needed to contextualize our findings with respect to normal versus atypical social brain development across childhood, adolescence, and adulthood.
